# DOES CHANGE IN COGSTATE MONTHLY COGNITIVE MONITORING OF OLDER ADULTS TRACK WITH 2-YEAR CHANGE IN GLOBAL COGNITION?

**DOI:** 10.1093/geroni/igz038.2409

**Published:** 2019-11-08

**Authors:** Taylor J Atkinson, Ross Andel

**Affiliations:** University of South Florida, Tampa, Florida, United States

## Abstract

CogState is a repeatable, accessible online cognitive testing suite with evidence of low practice effects that could be used as a monitoring tool to detect cognitive decline early. We compared participants’ change in CogState to change in the Telephone Interview for Cognitive Status (TICS). Participants (N = 41, age M = 75.5, 66% female) completed monthly CogState and two TICS assessments over two years. Reaction time on a psychomotor speed task, attention task, and working memory task, and accuracy on a memory task were assessed. A TICS difference score was calculated to measure change. Standardized scores were used. Covariates were age, sex, and education. Paired t-tests indicated that participants performed worse on the TICS the second time, p = .02, Cohen’s d = 0.39, but better on their last working memory task, p = .007, Cohen’s d = 0.45, and their last memory task, p = .001, Cohen’s d = 0.56. Growth curve models indicated CogState memory and working memory scores improved over time, ps < .05, by 0.17 SD accuracy units and 0.16 SD speed units, respectively. There were no significant TICS difference score by time interactions, indicating that changes in CogState were not related to change in TICS. CogState monthly repeat assessment did not track with change in the TICS, indicating that participants may become more proficient in task performance with repeated testing even while global cognition worsens. Despite prior evidence of low practice effects, less frequent assessment may still be warranted to avoid losing sensitivity to change.

